# Acute effects of naturalistic THC vs. CBD use on recognition memory: a preliminary study

**DOI:** 10.1186/s42238-020-00034-0

**Published:** 2020-09-17

**Authors:** Tim Curran, Hélène Devillez, Sophie L. YorkWilliams, L. Cinnamon Bidwell

**Affiliations:** 1 Department of Psychology and Neuroscience, UCB 345, University of Colorado Boulder, Boulder, CO 80309-0345 USA; 2 Department of Psychology and Neuroscience, University of Colorado Boulder, Boulder, CO 80309-0345 USA; 3 Department of Psychology and Neuroscience, Institute of Cognitive Science, University of Colorado Boulder, Boulder, CO 80309-0345 USA

**Keywords:** Marijuana, Cannabis, Cannabinoids, Episodic memory, Verbal memory

## Abstract

The ratio of ∆9-tetrahydrocannabinol (THC) to cannabidiol (CBD) varies widely across cannabis strains. CBD has opposite effects to THC on a variety of cognitive functions, including acute THC-induced memory impairments. However, additional data are needed, especially under naturalistic conditions with higher potency forms of cannabis, commonly available in legal markets. The goal of this study was to collect preliminary data on the acute effects of different THC:CBD ratios on memory testing in a brief verbal recognition task under naturalistic conditions, using legal-market Colorado dispensary products. Thirty-two regular cannabis users consumed cannabis of differing THC and CBD levels purchased from a dispensary and were assessed via blood draw and a verbal recognition memory test both before (pretest) and after (posttest) *ad libitum* home administration in a mobile laboratory. Memory accuracy decreased as post-use THC blood levels increased (*n* = 29), whereas performance showed no relationship to CBD blood levels. When controlling for post-use THC blood levels as a covariate, participants using primarily THC-based strains showed significantly worse memory accuracy post-use, whereas subjects using strains containing both THC and CBD showed no differences between pre- and post-use memory performance. Using a brief and sensitive verbal recognition task, our study demonstrated that naturalistic, acute THC use impairs memory in a dose dependent manner, whereas the combination of CBD and THC was not associated with impairment.

## Introduction

Cannabis produces acute memory impairment during intoxication (Bossong et al. 2014; Broyd et al. 2016; Lundqvist 2005; Ranganathan and D’Souza 2006), although regular users may not show these acute decrements in performance (Ranganathan and D’Souza 2006; Schoeler and Bhattacharyya 2013). Cannabis contains many cannabinoids that may have differential effects on memory. Overall, research studies have not sufficiently considered the fact that cannabis exists in different forms and have not characterized the effects of cannabis as the compound action of different cannabinoids that vary in terms of their pharmacological effects. Two of the primary cannabinoids, ∆9-tetrahydrocannabinol (THC) and cannabidiol (CBD), have some opposing effects (Osborne et al. 2017; Rømer Thomsen et al. 2017; Zhornitsky and Potvin 2012), and the ratio of THC to CBD varies dramatically among different strains of cannabis, with some strains in Colorado testing at greater than a 20:1 CBD to THC ratio, while other strains have a 1:1 THC to CBD ratio, and many have negligible amounts of CBD. Furthermore, most research to date has used low-strength government-grown cannabis (THC ranging from 3 to 6%) that lacks other key cannabinoids (CBD close to 0%) and has been administered in tightly controlled laboratory environments, all of which maximize internal validity, but compromise external validity. Currently, the THC strength of recreational cannabis in Colorado can exceed 25%, and the strength of CBD comes close to 25% in some strains (Vergara et al. 2017).

Recent reviews suggest that CBD has no effect on cognition in healthy individuals, but can improve cognitive processes including attention, executive function, working memory, and episodic memory in various pathological conditions including acute THC intoxication (Osborne et al. 2017; Rømer Thomsen et al. 2017; Zhornitsky and Potvin 2012). In this context, CBD has been considered as a potential treatment for cognitive impairments resulting from schizophrenia, Alzheimer’s disease, ischemia, inflammatory states, and hepatic encephalopathy (a disorder resulting from acute and chronic liver failure) (Osborne et al. 2017). Thus, a better understanding of the protective effects of CBD during THC impairment may also provide insights about CBD’s potential for improving cognitive problems with varying etiologies.

Previous episodic memory studies indicate that cannabinoids such as CBD may counteract the effects of THC. Chronic benefits of CBD were suggested in a study showing better recognition memory for words in regular cannabis users with CBD present in their hair (Morgan et al. 2012). A prior naturalistic study assessed acute effects in users who already prefer high-CBD strains (Morgan et al. 2010b). Prose recall was significantly higher after use of cannabis that was high in CBD compared to the low CBD group. Other previous studies have suggested that CBD acutely reduces THC-related learning and memory impairments in well-controlled human (Englund et al. 2013) and animal studies (Vann et al. 2008; Wright Jr. et al. 2013). In one clinical study, subjects were given an oral dose of CBD (600 mg) or a placebo 210 min ahead of an intravenous injection of THC (1.5 mg). Those in the CBD group showed better episodic memory (delayed free recall) compared to the placebo group (Englund et al. 2013). On the other hand, another prose recall study compared placebo, THC 8 mg, CBD 16 mg and THC 8 mg + CBD 16 mg in a randomized, double-blind crossover design with vaporizer inhalation (Morgan et al. 2018). Both the THC and THC + CBD conditions impaired memory, but CBD had no effects, even though the same subjects showed some protective effects of CBD in identification of facial emotions (Hindocha et al. 2015). These studies highlight that the effects of THC and CBD on memory may vary by dose, timing, and form of administration. Furthermore, they point to the need for measuring blood cannabinoid levels after cannabis administration to determine THC and CBD exposure.

In one preliminary study, we began to assess the effects of higher THC and CBD concentrations on verbal recall (Bidwell et al. 2018). Regular cannabis users were asked to use either a + THC/−CBD strain (~ 17% THC, < 1% CBD; *n* = 11) or a + THC/+CBD strain (8% THC, 16% CBD; *n* = 12) that was acquired from a local dispensary. Participants used the assigned cannabis strain in accordance with their normal usage habits for 3 days, including a final use on the third day. Immediately after this final use, participants were transported to the lab by the research team for a detailed assessment of its effects on neuro- and bio- behavioral functions, including memory. Blood draws were collected before the three-day use period (i.e., baseline), immediately upon arrival at the lab (within 15 min of last cannabis use), and at the end of the two-hour assessment in order to verify effective strain assignment and cannabinoid exposure. Testing included the International Shopping List Task (ISLT) as a measure of verbal recall (Thompson et al. 2011). The ISLT consists of a 12-item shopping list that was read out loud to the participant three times in the same order. After 30 min, a delayed free recall test was given. Results suggested that recall performance was negatively correlated with THC blood levels for the THC-only strain (+THC/−CBD), but recall performance was not significantly correlated with THC blood levels for the CBD-containing strain (+THC/+CBD). These preliminary findings suggest that the strain type differentially affected recall and prompt further research into the impacts of naturalistic administration of legal market THC and CBD on memory function.

The present experiment used a novel design to naturalistically assess the effects of real-world cannabis products on memory with the use of a mobile pharmacology and phlebotomy laboratory, which was driven to participants’ homes to allow assessment of participants both immediately before and after naturalistic administration of real-world cannabis. Although cannabis is legal at the state level in Colorado, researchers are not allowed to have participants use or handle state legal cannabis in any form on university property or in the presence of University staff, as this would be a violation of the federal Drug Free Schools Act. While we could have participants self-administer at home and take a taxi to the lab (a strategy we attempted in our prior work (Bidwell et al. 2018)), there are two major disadvantages of this approach: 1) We are unable to take a baseline assessment immediately prior to administration of an acute dose of cannabis, and 2) There is a high degree of variability in when participants actually arrive at the lab, meaning it is difficult to standardize assessments as a function of time since consumption. Using our mobile pharmacology and phlebotomy lab, we were able to draw blood to assess cannabinoid levels and collect assessments immediately before cannabis use, and at more precise time points post use. This innovative approach allows us to conduct cutting edge research on the acute effects of cannabis strains legally available in our state, but not allowed in University laboratories.

In the present experiment, we sought to collect feasibility data that would allow us to replicate and extend prior work using a mobile laboratory (Bidwell et al. 2018), facilitate more precise timing of pre- and post- cannabis use assessments, and administer a verbal recognition memory task. These feasibility data were collected in the context of two larger studies focused on the acute effects of high potency legal market forms of concentrate (State of Colorado Marijuana Research Grant 96,947 to LCB) or flower cannabis (R01DA039707 to Kent E Hutchison). The two studies were otherwise identical regarding the tasks that subjects completed. The detailed procedures and primary outcomes of these larger studies are described and reported elsewhere (Bidwell et al. 2020). A recognition memory task, which was not part of the original aims of either study, was selected to extend our previous ISLT results (Bidwell et al. 2018) beyond free recall with a task that provides better control over memory retrieval conditions (Kahana 2012). In addition to recollection processes required for free recall, recognition engages familiarity-based memory processes (Diana et al. 2006; Malmberg 2008; Yonelinas 2002) that we plan to dissociate in future cannabis studies with event related potentials (ERPs, Curran and Doyle 2011; Rugg and Curran 2007). Regular cannabis users twice completed a verbal recognition memory task with words: Before (“pretest”) and approximately 35 min after *ad libitum* use (“posttest”) of their assigned cannabis strain. Several strains of flower and two concentrates were used, and each strain fell into one of two groups: THC and THC + CBD (see Table 1). We assessed the effects of each cannabis strain as the degree of memory performance decrement from the pretest to the posttest. We hypothesized that CBD should have a protective effect on THC-induced memory impairment, so we predicted that the pre/post decrement would interact with strain such that the decrement would be largest in the THC group compared to the THC + CBD group. Furthermore, a blood draw taken immediately after cannabis consumption was used to quantify peak levels of THC and CBD. We predicted that posttest memory performance would decline as THC levels increased, and THC and CBD levels would interact such that THC levels would have diminished effects as CBD levels increased.

**Table 1:** Assignment of different products to groups

**Group**	**Form**	**% THC**	**% CBD**	**n** **Tested**	**n** **Analysis**^**a**^
**THC**				*n* = 15	
	Concentrate	90%	0%	5	
	Concentrate	70%	0%	4	
	Flower	24%	1%	6	
**THC + CBD**				*n* = 17	*n* = 16
	Flower	14%	9%	6	6
	Flower	6%	9%	4	3
	Flower	9%	10%	2	2
	Flower	1%	23%	5	5

^a^Details of exclusions are provided in the Results section

## Method

### Participants

Participants (32 cannabis users aged between 21 and 66 years) were recruited from the Boulder-Denver Metro area in Colorado using social media postings and mailed flyers. Because the goal was to collect feasibility data using a novel methodology, the recognition memory task reported here was only assessed in 32 subjects. Trained research staff screened eligible participants via telephone. Criteria for inclusion in the study were: 1) Aged between 21 and 70; 2) Used cannabis at least 4 times in the past month; 3) Experience with the highest potency of cannabis that could be assigned in the study (24% THC for flower groups and 90% THC for concentrate groups); 4) No other non-prescription drug use in the past 60 days; with a urine toxicology screen; 5) No daily tobacco use; 6) Reported drinking 2 times or fewer per week, and ≤ 3 drinks per occasion; 7) Not be pregnant, or trying to become pregnant; 8) No self-reported prior or current psychotic or bipolar disorder. Those eligible for the study completed both a baseline appointment and an experimental appointment, described in greater detail below.

### Procedure

#### Overview of Design of Feasibility Study

In an observational study, cannabis flower and concentrate users were assigned to purchase and use a legal market THC only or THC + CBD product. Participants completed a verbal recognition memory task at baseline and during an experimental mobile laboratory assessment approximately 50 min after *ad libitum* administration of their product. Thus, product strain was manipulated between participants and pre/post-use memory assessment was manipulated within participants.

#### Baseline appointment

Participants were instructed not to use cannabis on the day of their baseline appointment, which took place at the research team’s on-campus laboratory. After completing the informed consent process, a Breathalyzer (Intoximeter, Inc., St. Louis, MO) and urinalysis test was administered to ensure that participants had no alcohol, sedatives, cocaine, opiates, or amphetamines in their system. If either test was positive, the baseline appointment was rescheduled, and participants with repeated positives were terminated from the study. Female participants were required to take a urine pregnancy test, to ensure that they were not currently pregnant. Participants completed questionnaires on demographics, lifestyle, substance use, and medical history. After baseline questionnaires were completed, participants provided a blood draw.

Before leaving the baseline appointment, each participant was given a card with directions to a local dispensary in order to purchase their study product. Several strains of flower and two concentrates were used and randomly assigned in the larger studies (details on these procedures are in Bidwell et al. (2020)). In order to achieve a wide range of THC and CBD exposure for the purposes of this verbal recognition feasibility study, individuals were assigned to the full range of strains being tested in the parent studies and each strain was grouped into one of the following categories for the purposes of this feasibility study: THC or THC + CBD (see Table 1). Specifically, participants who primarily used cannabis concentrates purchased either a 70% or 90% THC concentrate which fell into the THC group. Participants who primarily used flower, instead of other cannabis products, were given instructions to purchase one of the following flower strains: 24% THC and 1% CBD, which fell into the THC group; or one of the THC + CBD group strains that contained either 14% THC and 9% CBD, 6% THC and 9% CBD, 9% THC and 10% CBD, or 24% CBD and 1% THC. The THC and CBD potency of each study product was tested and labeled consistent with State of Colorado requirements, in an International Organization for Standardization (ISO) 17,025 accredited laboratory. ISO 17025 is the highest recognized quality standard in the world for calibration and testing laboratories. Independent testing by University researchers is not permitted under federal law. Research staff were blinded to strain condition, and the blind was maintained by the dispensary and one senior member of the lab. The sample sizes of each group were: THC (*n* = 15) and THC + CBD (*n* = 17).

#### Experimental appointment

After participants obtained the study product, they were asked to use it exclusively, and *ad libitum*, for the 5 days leading up to the experimental appointment, which took place in a mobile laboratory outside of the participants’ place of residence. Participants were asked to abstain from using cannabis on the day of the appointment, prior to the experiment. At the first assessment of the day (pre-use), participants completed a blood draw and the primary outcome measures, followed by the first administration of the recognition task.^1^ Then they returned home to use their study cannabis *ad libitum* with their normally preferred method of administration. The THC group used 6 different administration methods: oil rig (*n* = 6), bong (*n* = 4), vaporizer (*n* = 1), glass straw (*n* = 2), joint (*n* = 1) and bubbler (*n* = 1). The THC + CBD group used 4 different administration methods: pipe (*n* = 7), bong (*n* = 5), vaporizer (*n* = 2) and joint (*n* = 2). Shortly thereafter, they returned to the mobile lab to complete the blood draw to estimate peak cannabinoid exposure, the primary outcome measures, and the recognition memory task again, while acutely intoxicated (acute post-use). The post-use recognition memory task took place 35 min after participants returned to the van.^2^

#### Measures

##### Past-month use of cannabis

To report on their typical use of cannabis at the baseline appointment, participants completed a calendar-assisted, researcher administered Timeline Followback that queried their use of alcohol, nicotine/tobacco, cannabis, prescription drugs, and illicit drugs over a 30-day retrospective timeframe (Dennis et al. 2004).

##### Cannabinoid content

Because University research staff are not permitted to handle legal market cannabis, we asked participants to weigh their product with a study-provided scale [American Weigh Scale, Gemini Series Precision Digital Milligram Scale (GEMINI-20)] at the experimental appointment both before and after *ab libitum* use. Although blood THC, CBD, and metabolite measures remain our primary measure of individual cannabinoid exposure, the weight that each *participant provided* (mg) was used to further estimate the amount of each cannabinoid consumed based on the percentages of THC and CBD contained in their specific study strain. While these mg estimates are not considered a primary measurement of cannabinoid dose, we include these data in order to facilitate integration and interpretation of our findings with prior controlled laboratory studies.

##### Blood cannabinoids

A certified phlebotomist collected 32 mL (2 tablespoons) of blood through venipuncture of a peripheral arm vein using standard, sterile phlebotomy techniques in order to assess plasma cannabinoids. Plasma was separated from erythrocytes by centrifugation at 400 xg for 15 min, transferred to a fresh microcentrifuge tube, and stored at − 80 °C. Plasma samples were sent to iC42 Clinical Research and Development (Department of Anesthesiology) on the Anschutz Medical Campus at the University of Colorado Denver. Four cannabinoids were quantified in the blood (THC and its primary metabolites THC-COOH and 11-OH-THC, and CBD) using validated high performance liquid chromatography/mass-spectroscopy (HPLC-MS/MS) (API5500) in MRM mode (Klawitter et al. 2017).

##### Recognition memory task

**Fig. 1:**
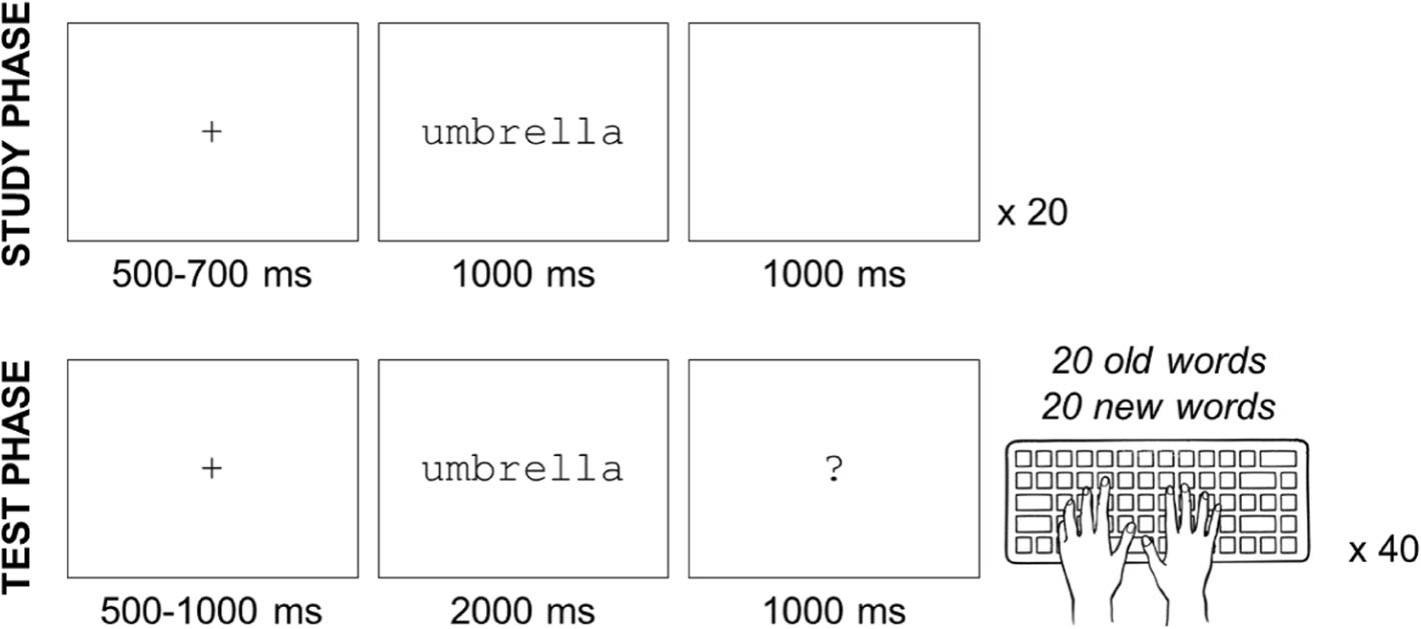
Time course of one trial during the study phase and the test phase

Figure 1 provides an overview of the recognition memory task procedures. In each of the two runs of the recognition memory task, subjects studied 20 words followed by a recognition memory test with 20 old (studied) and 20 new (non-studied) concrete nouns. The pretest and posttest tasks included different words, and the exact same lists were used for each participant to minimize variability. The four lists (2 old × 2 new) were matched on word length and Kucera-Francis written frequency (Kucera and Francis 1967). The study lists also included 2-word, non-tested buffer items at the beginning and end of the list to reduce primacy and recency effects. Each study trial started with a 500–700 ms fixation cross, followed by a word for 1000 ms, and ending with a 1000 ms blank screen. Participants were instructed to try to remember each word in preparation for the upcoming test. Participants played Sudoku for 3 min between each of the study and test lists to provide a distracting stimulus that would minimize active rehearsal during the delay. Each test trial started with a 500–1000 ms fixation cross, followed by a word for 2000 ms, and ending with a 1000 ms blank screen. Subjects were instructed to judge each word as old or new as quickly and accurately as possible, by pressing either a leftward (R or F) or a rightward (U or J) key on the keyboard. Assignment of response keys and left/right to old/new responses was counterbalanced across subjects.

## Analysis

### Cannabinoid content

Cannabinoid plasma biomarker levels taken immediately post-use were our primary assessment of the strength of the effects of each cannabinoid, but cannabinoid content weight is also reported to facilitate comparison with other studies. The total weight of the product that each participant used was measured as the difference between pre- and post-use weight (mg Total, Table 2). The amount of each cannabinoid consumed by each participant was estimated by multiplying the total weight used by the percentage of THC and CBD in that subject’s strain (mg THC and mg CBD, Table 2). To examine differences in cannabinoid content across groups, analyses were performed in a mixed-design ANOVA with cannabinoid type (CBD, THC) as a within-subject factor and strain group (THC, THC + CBD) as a between-subject factor.

**Table 2:** Participant characteristics and blood biomarkers by strain group. Means are reported with 95% confidence intervals in brackets

	Strain group		***t*** test
	THC	THC + CBD	
***N***	15	16	*–*
**Age**	26.80 [24.13–30.07]	32.81 [26.37–43.94]	*ns*
**Gender** **(#F)**	7	8	*–*
**First age of regular cannabis use**	16.15 [14.46–17.38]^a^	21.44 [18.37–31.69]	*ns*
**Cannabis days in past 30 days**	25.15 [21.01–27.38] ^a^	18.94 [14.68–23.00]	*
**Time away from the van**	14.13 [10.87–19.44]	17.12 [14.06–21.50]	*ns*
**Cannabinoid content (mg**)			
**CBD**	0.84 [0.26–1.77] ^b^	41.79 [25.71–79.28]	**
**THC**	86.26 [57.87–124.37] ^b^	20.06 [12.24–33.54]	**
**Total**	163.79 [108.86–231.05] ^b^	282.06 [200.95–415.31]	*ns*
**Blood plasma (ng/m**L)			
**Pretest**			
**CBD**	0.43 [0.16–0.95]	1.21 [0.68–2.56]	*ns*
**THC**	9.17 [5.11–16.44]	2.75 [1.53–4.30]	*
**THC-COOH**	67.50 [41.97–99.44]	41.78 [22.69–75.50]	*ns*
**11-OH-THC**	9.28 [5.36–17.22]	5.43 [2.80–9.74]	*ns*
**Sum** **THC +** **metabolites**	85.96 [54.27–131.35]	49.96 [27.33–85.11]	*ns*
**Posttest**			
**CBD**	1.15 [0.56–2.68]	35.25 [19.88–68.81]	**
**THC**	278.31 [144.75–684.41]	64.21 [36.46–109.80]	*ns*
**THC-COOH**	75.22 [49.04–111.51]	40.54 [23.04–62.59]	*ns*
**11-OH-THC**	6.27 [3.27–13.65]	3.12 [1.45–7.65]	*ns*
**Sum** **THC + metabolites**	359.79 [211.78–810.10]	107.87 [67.23–155.49]	*

The right column indicates significant differences from the *t* test (*: *p* < .05, **: *p* < .01)

^a^ indicates *n* = 13 because two THC participants did not complete the questionnaires

^b^ indicates *n* = 14 because one THC participant did not weigh her or his product

### Cannabinoid plasma biomarker levels

Given that our observational study involved *ad libitum* use of various cannabis products, cannabinoid plasma biomarker levels obtained from blood taken immediately after cannabis administration were our primary quantitative assessment of individual exposure to each relevant cannabinoid. As shown in Table 2, four cannabinoids were quantified in the blood (THC and its primary metabolites THC-COOH and 11-OH-THC, and CBD). Analysis of THC levels were performed with a composite THC + metabolites measure, which is the sum of the three THC levels. These measurements were analyzed in a mixed-design ANOVA with session (pretest, posttest) and cannabinoid type (CBD, sum THC + metabolites) as within-subject factors and strain group (THC, THC + CBD) as a between-subject factor.

### Estimated cannabis dose and strain effects on memory

As is typical in recognition memory research (Macmillan and Creelman 2005; Malmberg 2008; Neath and Surprenant 2003; Wixted 2007) and consistent with previous studies on the effects of THC and CBD on recognition memory (Morgan et al. 2012; Morgan et al. 2010b), *d’* (accuracy in discriminating old vs. new words) was used as the primary measure of memory performance. The hit rate (*H*, proportion of correct “old” responses to studied words) and false alarm rate (*FA*, proportion of incorrect “old” responses to non-studied words) are used to calculate *d’* (*d′ = z*_*H*_ *− z*_*FA*_*,* where *z* is the standard normal distribution). Given the distribution of the metabolites, we performed a log transformation of the metabolite data.

For *d’* we first ran a regression model to examine how cannabinoid levels (sum THC + metabolites and CBD) were associated with accuracy (*d’*).

1 $$ \mathrm{d}{\hbox{'}}_{\mathrm{i}}={\upbeta}_0+{\upbeta}_1\log \left({\mathrm{THCLevel}}_{\mathrm{i}}\right)+{\upbeta}_2\log \left({\mathrm{CBDLevel}}_{\mathrm{i}}\right)+{\upbeta}_3\log \left({\mathrm{THCLevel}}_{\mathrm{i}}\right)\ast \log \left({\mathrm{CBDLevel}}_{\mathrm{i}}\right)+{\upvarepsilon}_{\mathrm{i}} $$

The regression allows us to assess how memory accuracy was affected by differences in the strength of neurophysiological exposure to each cannabinoid alone and in combination. Second, the effect of strain group on memory accuracy (*d’*) was analyzed in a mixed-design analysis of variance (ANOVA) with session (pretest, posttest) as a within-subject factor and strain group (THC and THC + CBD) as a between-subject factor. Because the THC content was lower in the product consumed by the THC + CBD group, we ran a second ANOVA with log (THC + metabolites) as a covariate in this ANOVA.

Our primary measure of recognition memory performance was d’, but Table 3 shows other performance measures for completeness*,* including the hit and false alarm rates used to calculate *d’*. Table 3 shows a measure of response bias (*c* = − 1/2 * [*z*_*H*_ *− z*_*FA*_]), where negative values indicate a liberal bias to respond “old” and positive values indicate a conservative bias to respond “new”. Table 3 also shows response time (RT). Each of these performance measures were separately analyzed in a mixed-design analysis of variance (ANOVA) with session (pretest, posttest) as a within-subject factor, strain group (THC and THC + CBD) as a between-subject factor, and THC + metabolite levels as a covariate.

**Table 3:** d’, hit rate, FA (false alarm) rate, *c* (response bias) and reaction time (RT) for pre- and posttest, for the two strain groups, THC and THC + CBD. Means are reported with 95% within subject confidence intervals in brackets. The right columns indicate significant differences from the t-test on group differences (*: *p* < .05, **: *p* < .01)

	Strain group		***t*** test
	THC	THC + CBD	
**Pretest**			
**d’**	1.62 [1.25–1.94]	1.93 [1.48–2.39]	*ns*
**Hit rate**	0.72 [0.64–0.78]	0.77 [0.68–0.85]	*ns*
**FA** **rate**	0.21 [0.14–0.29]	0.17 [0.13–0.22]	*ns*
**Response Bias**	0.16 [−0.04–0.34]	0.07 [−0.06–0.24]	*ns*
**RT (s)**	0.83 [0.78–0.90]	0.91 [0.83–1.01]	*ns*
**Posttest**			
**d’**	0.84 [0.55–1.27]	1.99 [1.41–2.55]	**
**Hit rate**	0.65 [0.52–0.74]	0.82 [0.74–0.89]	*
**FA** **rate**	0.38 [0.28–0.45]	0.23 [0.15–0.34]	*
**Response bias**	−0.03 [− 0.24–0.41]	−0.10 [− 0.24–0.04]	*ns*
**RT (s)**	0.92 [0.83–1.07]	0.89 [0.79–1.02]	*ns*

Multiple comparisons were assessed with Bonferroni post-hoc tests (with corresponding *p*-values reported as *p*_*bf*_) for all analyses.

## Results

One of the 32 participants was excluded from analyses because their pretest blood levels exceeded mean + 3 standard deviation over all participants, when considering the combination of THC + metabolites level (sum of THC, THC-COOH and 11-OH-THC) and CBD level.^3^ This reduced the THC + CBD strain group from 17 to 16 participants (see Table 1).

As seen in Table 2, the strain groups did not significantly differ in age, first age of regular cannabis use, or time away from the van. We did observe a significant difference in cannabis consumption for the past 30 days, showing more cannabis use in the THC group compared to the THC + CBD group.

### Cannabinoid content

One participant did not weigh her or his product, so dosage results are based on only 14 subjects in the THC group. As reported in Table 2, the groups did not differ significantly in the total amount (mg) of product they consumed during at-home administration. However, they did differ in the amount (mg) of CBD and THC. As expected based on product content and group assignment, and as shown in Table 2, results indicated that each group differed on THC and CBD dosages in the expected directions. The THC group had the highest THC doses and the CBD group had the highest CBD doses.

### Cannabinoid plasma biomarker levels

Cannabinoid plasma biomarker levels (Table 2) were analyzed in a mixed-design ANOVA with 2 sessions (pretest, posttest) and 2 cannabinoid types (CBD, sum THC + metabolites) as within-subject factors, and strain (THC, THC + CBD) as a between-subject factor. Pre-test THC levels fell < 10 ng/mL on average across both groups, supporting that participants complied with day of abstinence procedures prior to their mobile laboratory study appointment.

Analysis of cannabinoid plasma biomarker levels revealed a main effect of session, *F*(1,29) = 11.44, *p* < .001, ${\eta}_p^2$ = 0.28, and a significant main effect of cannabinoid type, *F*(1,29) = 16.12, *p* < .001, ${\eta}_p^2$ = 0.36. Cannabinoid type interacted with strain group, *F*(1,29) = 5.25, *p* < .05, ${\eta}_p^2$ = 0.15, showing that sum THC + metabolite levels were higher for the THC group compared to the THC + CBD group (*p*_*bf*_ < .05). Cannabinoid type interacted with session, *F*(1,29) = 7.69, *p* < .01, ${\eta}_p^2$ = 0.21, showing that the level of sum THC + metabolites was higher at posttest (i.e., after cannabis use) compared to pretest (*p*_*bf*_ < .001). There was a significant 3-way interaction between cannabinoid type, strain group, and session, *F*(1,29) = 5.42, *p* < .05, ${\eta}_p^2$ = 0.16. When this interaction was decomposed with Bonferroni-corrected post hoc tests, they indicated that the strain groups did not differ on any pretest levels, but posttest sum THC + metabolites levels were higher for the THC group than the THC + CBD group (*p*_*bf*_ < .001). When testing each measure separately (Table 2), we only observed a significant difference for THC levels at pretest. Posttest CBD levels were higher for the THC + CBD group than the THC group, whereas posttest THC levels and sum THC + metabolites were higher for the THC group than the THC + CBD group.

### Cannabis dose and strain effects on memory

**Fig. 2:**
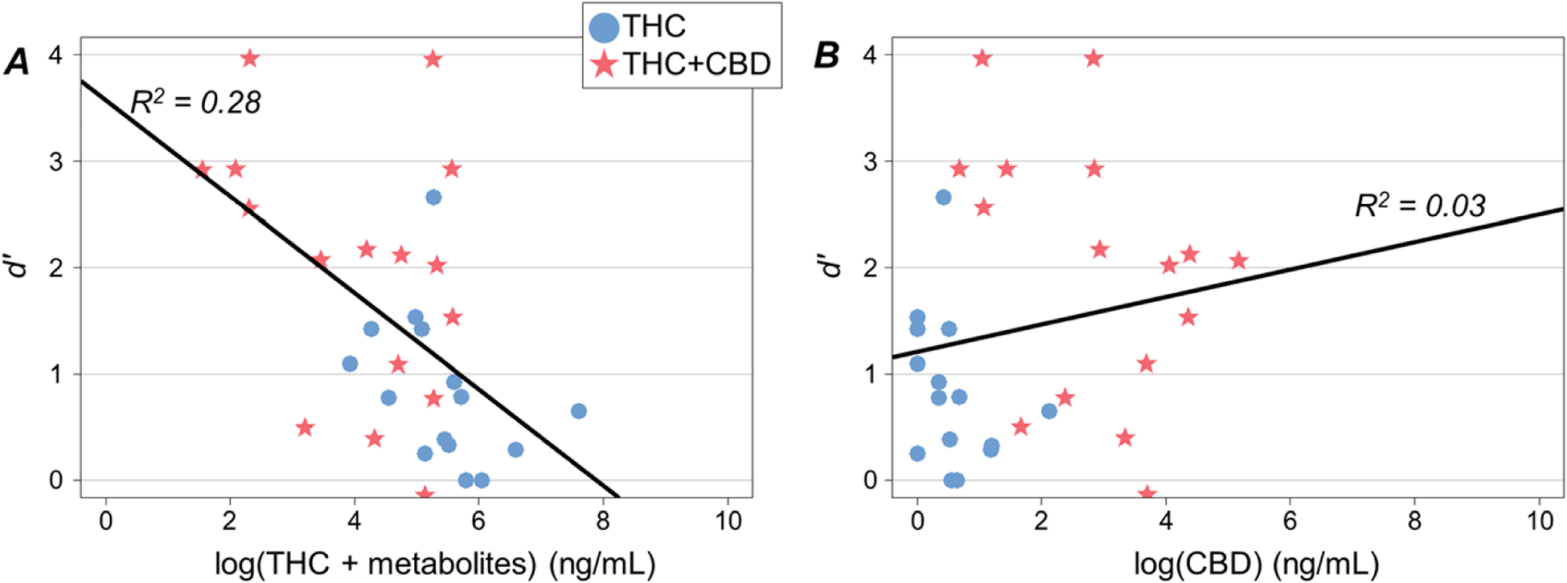
Accuracy d’ according to blood biomarkers log (THC + metabolites) (**a**) and log (CBD) (**b**) during posttest, for the two strain groups: THC and THC + CBD. The black lines represent the correlation between accuracy and blood biomakers with R^2^ reported

First, we ran a regression model (Eq. 1) to examine how cannabinoid levels (THC + metabolites and CBD) were associated with accuracy (d’). The model revealed that the level of THC + metabolites was significantly negatively correlated to accuracy (*p* < .05, ${\eta}_p^2$ = 0.28) (Fig. 2a), but neither the effect of CBD (Fig. 2b) nor the THC × CBD interaction was significant. This result was observed across the two strain groups, and neither THC nor CBD blood levels were significantly correlated with *d*′ within each strain group.

**Fig. 3:**
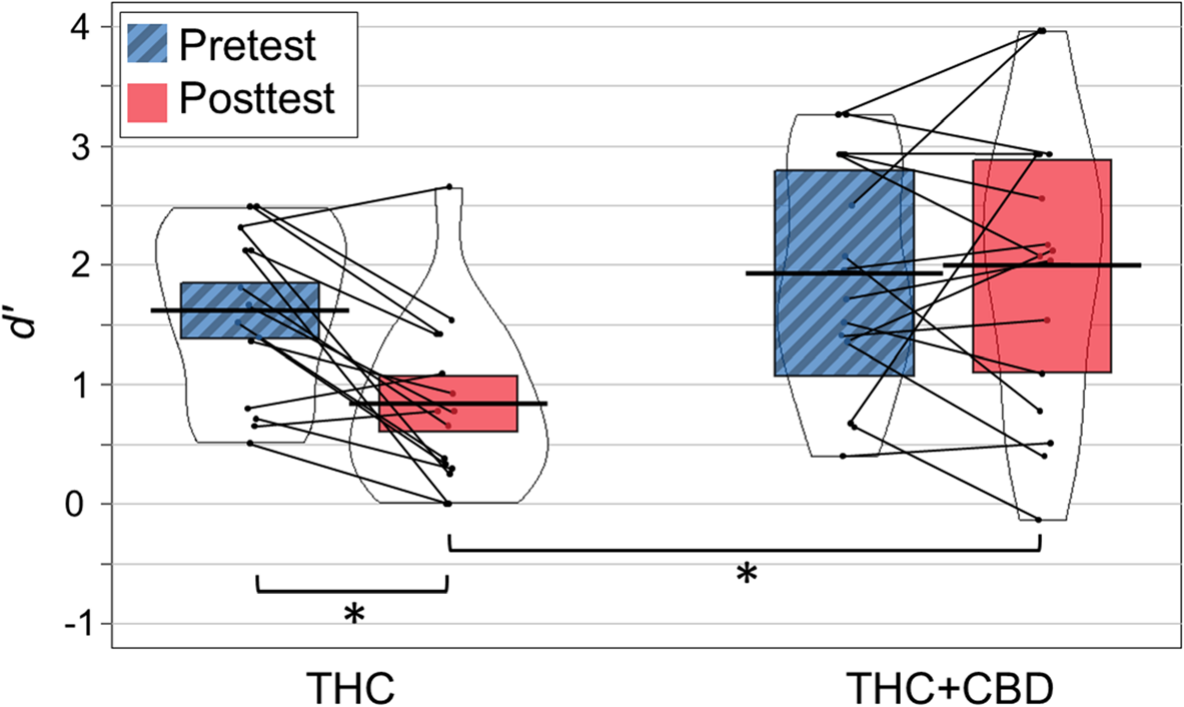
Accuracy *d*′ for pretest and posttest, for the two strain groups: THC and THC + CBD. Colored regions represent the 95% within subject confidence intervals (Morey 2008). Thick black lines represent the mean. Individual data points represent the mean *d’* for each participant. Thin black lines connect individuals across conditions. Asterisks show results of the Bonferroni post-hoc tests (* *p*_*bf*_ < 0.05)

Second, accuracy (*d*′, Fig. 3) was analyzed in a mixed-design analysis of variance (ANOVA) with session (pretest, posttest) as a within-subject factor, and strain group (THC, THC + CBD) as a between-subject factor. *d*′ significantly decreased between pre- and post-test, *F*(1, 29) = 5.84, *p* < .05, ${\eta}_p^2$ = 0.17, and *d*^′^ was significantly higher for the THC + CBD group compared to the THC group, *F*(1, 29) = 6.05, *p* < .05, ${\eta}_p^2$ = 0.17. The significant session × strain group interaction, *F*(1,29) = 7.90, *p* < .01, ${\eta}_p^2$ = 0.21, showed that accuracy was lower at posttest than pretest for the THC group (*p*_*bf*_ < .01), but not for the THC + CBD group. We also observed that the accuracy at posttest was lower for the THC group than for the THC + CBD group (*p*_*bf*_ < 0.01). Additionally, sum THC + metabolite blood plasma levels were included as a covariate since it significantly predicted memory accuracy in the regression analysis and because the THC content of the product consumed by the THC + CBD group was lower in THC. As performed in previous analyses, we used the log transform of metabolite data. The covariate log (THC) was significant, *F*(1,28) = 7.79, *p* < .01, ${\eta}_p^2$ = 0.22. The significant session × strain group interaction, *F*(1,28) = 6.18, *p* < .05, ${\eta}_p^2$ = 0.18, showed similar results as before, with lower accuracy at posttest compared to pretest for the THC group (*p*_*bf*_ < .01), but not for the THC + CBD group. Also, the accuracy at posttest for the THC group was lower than for the THC + CBD group (*p*_*bf*_ < 0.01).

Consistent with our approach for *d’*, each of the other performance measures was separately analyzed in a mixed-design analysis of variance (ANOVA) with session (pretest, posttest) as a within-subject factor, and strain (THC, THC + CBD) as a between-subject factor. Results are presented without a covariate. When adding log (THC) as a covariate, no significant effects were observed for the 4 measures. Analysis of false alarm (FA) rate indicated a significant main effect of session, *F*(1, 29) = 18.45, *p* < .001, ${\eta}_p^2$ = 0.39, showing a higher rate of FA at posttest compared to pretest. Session also interacted with strain for FA, *F*(1, 29) = 4.86, *p* < .05, ${\eta}_p^2$ = 0.14, such that only the posttest FA rate was higher for the THC group than for the THC + CBD group (Table 3). Analysis of response bias (*c*) indicated a significant effect of session, *F*(1, 29) = 5.79, *p* < .05, ${\eta}_p^2$ = 0.17, such that subjects were somewhat conservative pretest (tended to respond “no” more than “yes”) but somewhat liberal posttest (tended to respond “yes” more than “no”). Analysis of hit rate and reaction time revealed no significant effects. The presence of significant posttest hit rate effects in the *t* tests (Table 3), but not in the ANOVA, suggests that ANOVA did not have sufficient power to detect the session × strain interaction for this outcome.

## Discussion

This study demonstrates the feasibility of a brief and mobile verbal recognition memory task for naturalistic and experimental studies of the acute effects of cannabis. Participants completed a recognition memory task before (pretest) and shortly after (posttest) *ad libitum* acute administration of cannabis products with varying THC:CBD ratios. Participants using products containing primarily THC showed significantly worse memory accuracy (*d’*) after use than before use, whereas subjects using strains containing both THC and CBD showed no differences between pre- and posttest memory performance. When blood cannabinoid levels were considered, *d’* was negatively correlated with THC levels, whereas performance showed no association with CBD levels. Thus, acute THC use was associated with impaired memory in a dose dependent manner, whereas the combination of THC and CBD was not associated with impaired memory.

Compared to other recent studies examining the acute effects of THC on episodic memory, the present study included more naturalistic methods of cannabis use and higher dosage. Recognition accuracy was better before than after THC consumption and decreased as THC blood levels increased. Our participants self-administered their assigned products *ad libitum* using their normally preferred methods at home. The mean estimated THC dosage across both the THC and THC + CBD strain groups was 58.61 mg (range = 1.92–235.8 mg). In a broad review of studies of cannabis use on human cognition from 2004 to 2015, Broyd et al. (2016) identified 11 studies investigating acute effects on verbal episodic memory. Of those demonstrating acute memory deficits, five administered intravenous (IV) THC (D'Souza et al. 2004; D’Souza et al. 2008; Englund et al. 2013; Morrison et al. 2009; Ranganathan et al. 2012), two administered vaporized cannabis (Liem-Moolenaar et al. 2010; Theunissen et al. 2015), and one administered oral THC (nabilone) (Wesnes et al. 2009). Dosage in these studies ranged from 2 to 12 mg of THC. More recent studies have documented episodic memory impairments after acute use of 8 mg of THC with a vaporizer (Morgan et al. 2018) and 10.73 mg of THC with experimenter-regimented joint smoking (Hindocha et al. 2015). Thus, we have replicated prior work under more naturalistic conditions and higher doses, as well as replicating our previous free recall results in a separate sample of participants with a recognition memory task (Bidwell et al. 2018).

As predicted, the deleterious effects of THC on recognition memory accuracy were not present when CBD was co-self-administered. Because THC levels were negatively correlated with posttest memory accuracy and THC levels differed between strain groups, we controlled for THC levels as a covariate and found a significant interaction between strain group and pre/posttest sessions. Participants using products that contained only THC showed memory accuracy decrements from pre- to posttest. No such decrements were observed in subjects using both THC and CBD. While preliminary, this finding is generally consistent with other suggestions that CBD and THC can have opposing effects on a variety of outcomes (Bidwell et al. 2018; Osborne et al. 2017; Rømer Thomsen et al. 2017; Zhornitsky and Potvin 2012) as well as other recent episodic memory studies suggesting that CBD can counteract memory impairments caused by acute THC use (Bidwell et al. 2018; Englund et al. 2013; Morgan et al. 2010a; Morgan et al. 2010b). These prior studies have all used free recall measures of memory, which the present results extend to recognition memory. Both recollection and familiarity processes are thought to contribute to recognition memory, whereas only recollection is relevant to free recall (Diana et al. 2006; Malmberg 2008; Yonelinas 2002). Some older studies have suggested that acute cannabis use impairs recollection more than familiarity (Fletcher and Honey 2006; Ilan et al. 2004), but none have examined differential acute effects of THC vs. CBD. ERPs have proven useful for discriminating these processes (Curran and Doyle 2011; Rugg and Curran 2007) and we plan to use ERPs in future research examining THC and CBD effects on recognition memory.

In addition to being a small feasibility study that needs to be replicated, there are three primary limitations of the present study. First, like Morgan et al. (2010a, 2010b), assignment of subjects to strains was not completely random, so pre-existing differences between participants could have influenced the results. For example, regular users of high potency THC concentrates may be more or less susceptible to its acute effects than other subjects. Bidwell et al. (2018) and Englund et al. (2013) used random assignment, but only Bidwell et al. (2018) used naturalistic administration. Second, the 50 min that elapsed after consumption prior to the memory assessment (which occurred ~ 35 min after blood draw to assess peak cannabinoid levels) may have limited the observed effects of THC and CBD. On the other hand, we have found the effects of THC on verbal recall memory to be relatively persistent when international shopping list test (ISLT) performance was compared between 15 and 30 min after use versus 60–75 min after use (Bidwell et al. 2020). Third, given the nature of this observational pilot study we were not powered to include all relevant covariates or ethically able to match the groups on important characteristics such as cannabis use history, preferred form of cannabis (e.g. flower vs. concentrate), or preferred route of inhaled administration (e.g. bong, pipe, etc.). Furthermore, compared to the THC group, the THC + CBD group tended to be older (with age also ranging more widely), started regular cannabis use later, used less cannabis in the past month, and consumed significantly less THC in their assigned strain. Although the first three demographic trends were not significant, that may be attributable to the small sample size, so these factors could have contributed to group differences on memory. Despite these concerns, our strongest memory effects were shown in the THC group, which had the heaviest levels of use prior to the study sessions mitigating a concern that our findings are driven by tolerance effects in heavy users. Typically, heavier users are less likely to show acute decrements in memory performance (Ranganathan and D’Souza 2006; Schoeler and Bhattacharyya 2013).

### Summary

This study puts forward novel, naturalistic data on the feasibility of a brief and mobile recognition memory task that can assess the impacts of higher potency legal market forms of cannabis that vary in levels of THC and CBD. With an emphasis on external validity, we demonstrate the feasibility of a method for assessing cannabis-related memory impairment after the use of legal market forms of cannabis either in the field or in clinical settings. Very few studies have examined the cognitive effects of legal market cannabis, which leaves a gap in the current literature in regards to real world consumption patterns when legal market access as well as medical and recreational use is rapidly increasing. These findings contribute naturalistic data to the public health sphere on the impact of THC and CBD on memory function and are relevant to patients, medical providers, policy makers, and law enforcement.

## Data Availability

The data are available on the Open Science Framework (https://osf.io/x4yns/?view_only=7e3c4c3de122454c816893a47263e513).

## Abbreviations

11-OH-THC: 11-Hydroxy-THC, primary active metabolite of THC
ANOVA: Analysis of variance
c: Response bias
CBD: Cannabidiol
d’: Discriminability
ERP: Event related potential
FA: False alarm rate
H: Hit rate
HPLC-MS/MS: High performance liquid chromatography/mass-spectroscopy
ISLT: International Shopping List Task
ISO: International Organization for Standardization
IV: Intravenous
mg: Milligram
mL: Milliliter
MRM: Multiple reaction monitoring
ms: Millisecond
N, n: Sample size
ng/mL: Nano grams per milliliter
RT: Response time
SD: Standard deviation
THC: ∆9-tetrahydrocannabinol
THC-COOH: tetrahydrocannabinol carboxylic acid, major inactive metabolite of THC
xg: Times gravity
z: Standard normal distribution

## Footnotes

1 Because the recognition task was added onto another ongoing protocol, it was always run after the primary outcome measures for the main study which included assessments of other memory tasks, attention, inhibitory control, balance, and subjective drug effects. These tasks are unlikely to interfere with recognition memory results. The only other verbal memory test included was the International Shopping List Task (ISLT), which used different words than the recognition task. Our larger study found that THC administration was negatively associated with ISLT performance, but CBD results await ongoing data collection and analysis (Bidwell et al. 2020).

2 We do not have the specific time point for the memory assessment for each participant, so the time given here is an estimate based on the general flow of the protocol. The timing of the protocol should not differ between participants.

3 Results obtained without excluding the outlier were similar and are not presented in detail. In particular, *d*′ negatively correlated with THC blood levels (*p* < .05), but not CBD blood levels. The session × strain interaction on *d*′ was significant, with or without the log(THC) covariate (both *p* < .01).
